# *DM–AHR*: A Self-Supervised Conditional Diffusion Model for AI-Generated Hairless Imaging for Enhanced Skin Diagnosis Applications

**DOI:** 10.3390/cancers16172947

**Published:** 2024-08-23

**Authors:** Bilel Benjdira, Anas M. Ali, Anis Koubaa, Adel Ammar, Wadii Boulila

**Affiliations:** 1 Robotics and Internet-of-Things Laboratory, Prince Sultan University, Riyadh 11586, Saudi Arabia; aaboessa@psu.edu.sa (A.M.A.); akoubaa@psu.edu.sa (A.K.); aammar@psu.edu.sa (A.A.); wboulila@psu.edu.sa (W.B.); 2 SE & ICT Laboratory, LR18ES44, ENICarthage, University of Carthage, Tunis 1054, Tunisia; 3 Department of Electronics and Electrical Communications Engineering, Faculty of Electronic Engineering, Menoufia University, Menouf 32952, Egypt; 4 RIADI Laboratory, University of Manouba, Manouba 2010, Tunisia

**Keywords:** skin diagnosis consumer applications, healthcare image generation, healthcare image enhancement, melanoma detection, diffusion models, skin medical images, automatic skin hair removal, self-supervised techniques

## Abstract

**Simple Summary:**

Skin diseases can be serious, and early detection is key to effective treatment. Unfortunately, the quality of images used to diagnose these diseases often suffers due to interference from hair, making accurate diagnosis challenging. This research introduces a novel technology, the *DM–AHR*, a self-supervised conditional diffusion model designed specifically to generate clear, hairless images for better skin disease diagnosis. Our work not only presents a new, advanced model that expertly identifies and removes hair from dermoscopic images but also introduces a specialized dataset, DERMAHAIR, to further research and improve diagnostic processes. The enhancements in image quality provided by *DM–AHR* significantly improve the accuracy of skin disease diagnoses, and it promises to be a valuable tool in medical imaging.

**Abstract:**

Accurate skin diagnosis through end-user applications is important for early detection and cure of severe skin diseases. However, the low quality of dermoscopic images hampers this mission, especially with the presence of hair on these kinds of images. This paper introduces *DM–AHR*, a novel, self-supervised conditional diffusion model designed specifically for the automatic generation of hairless dermoscopic images to improve the quality of skin diagnosis applications. The current research contributes in three significant ways to the field of dermatologic imaging. First, we develop a customized diffusion model that adeptly differentiates between hair and skin features. Second, we pioneer a novel self-supervised learning strategy that is specifically tailored to optimize performance for hairless imaging. Third, we introduce a new dataset, named *DERMAHAIR* (DERMatologic Automatic HAIR Removal Dataset), that is designed to advance and benchmark research in this specialized domain. These contributions significantly enhance the clarity of dermoscopic images, improving the accuracy of skin diagnosis procedures. We elaborate on the architecture of *DM–AHR* and demonstrate its effective performance in removing hair while preserving critical details of skin lesions. Our results show an enhancement in the accuracy of skin lesion analysis when compared to existing techniques. Given its robust performance, *DM–AHR* holds considerable promise for broader application in medical image enhancement.

## 1. Introduction

Global skin cancer rates are escalating, highlighting the urgent need for accurate and efficient diagnostic methods. The World Health Organization (WHO) reports that skin cancers comprise a significant proportion of all cancer cases worldwide, with melanoma recognized as the most lethal form. The incidence of melanoma has been steadily increasing over the past few decades, marking it as a critical public health concern (WHO, 2021) [1]. Early detection is paramount in the fight against skin cancer, as it significantly improves prognosis. As part of this fight, there has been an increase in end user applications designed to diagnose skin health conditions. However, these applications are still hampered by the presence of many conditions that decrease the detection accuracy.

The integration of artificial intelligence (AI) [2,3,4,5,6] in medical imaging, particularly in dermatology, has greatly benefited the early detection and diagnosis of skin cancers, including melanoma [7,8,9]. Dermoscopy, a noninvasive skin imaging technique, is crucial in this process. However, the presence of hair in dermoscopic images often poses significant challenges [10], as it can obscure critical details of skin lesions and may lead to potential diagnostic errors. Physical removal of hair is a solution, but it can be uncomfortable for patients and may not always be feasible. To address this issue, we introduce *DM–AHR*, a novel conditional diffusion model specifically designed to generate hairless dermoscopic images that are more suitable for diagnosis and detection. Diffusion models have emerged as powerful tools in the field of machine learning, particularly for image-generation tasks. These models create high-quality, detailed images by iteratively refining a signal from a random noise distribution based on stochastic process principles. This approach allows for the generation of images with remarkable fidelity and diversity. Our research focuses on improving the quality of dermoscopic images, which is vital for early skin cancer detection and diagnosis. We believe *DM–AHR* can significantly contribute to this field.

The flexibility and robustness of diffusion models make them especially effective for tasks requiring nuanced understanding and manipulation of visual data. Our study repurposes diffusion models to enhance the clarity of medical skin images. The *DM–AHR* model is designed to discriminate between hair and underlying skin features, gradually generating a hair-free image from the original hairy image. This approach not only augments the visibility of critical dermatological details but also streamlines the pre-diagnostic process by eliminating the need for physical hair removal. The main contributions of this paper are as follows:

- We introduce *DM–AHR*, a customized self-supervised diffusion model for automatic hair removal in dermoscopic images. This model enhances the clarity and diagnostic quality of skin images by effectively differentiating hair from skin features.
- We demonstrate significant improvement in skin lesion detection accuracy after applying *DM–AHR* as a preprocessing step before the classification model.
- We introduce the novel DERMAHAIR dataset, advancing research in the task of automatic hair removal from skin images.
- We introduce a new self-supervised technique specifically tailored for the task of automatic hair removal from skin images.
- We demonstrate *DM–AHR*’s potential in broader skin diagnosis applications and automated dermatological systems.

This study details the architecture and optimization of *DM–AHR*. The structure of the paper is as follows: Section 2 discusses related works in the field, providing background and context for the current research. Section 3 describes the proposed methodology, detailing the design, implementation, and theoretical foundation of the *DM–AHR* model. Section 4 presents the experimental results, offering a thorough analysis of the model’s performance, comparisons with existing methods, and insights gained from the experiments. Finally, Section 5 concludes the paper, summarizing the key findings, discussing implications, and suggesting directions for future research.

## 2. Related Works

The domain of medical image analysis has seen substantial advancements due to the integration of AI and machine learning techniques. Particularly, in dermatology, the automatic analysis of dermoscopic images plays a crucial role in the early detection of skin diseases, including melanoma. This section reviews the existing literature in three primary areas: diffusion models in image processing, automatic hair removal techniques in dermoscopic images, and their implications for skin disease diagnosis.

First, diffusion models have gained prominence in image processing due to their exceptional ability to generate and reconstruct high-quality images. Recent studies, such as those by [11,12], have demonstrated the potential of these models in tasks like image denoising, super-resolution, and inpainting. Their application in medical image analysis, however, remains relatively unexplored. The adaptability of diffusion models to different contexts, as shown by [13], provides a foundation for their application in enhancing the quality of medical images, including dermoscopic images. Another notable development in the domain of dermatoscopic image enhancement is the Derm-T2IM approach, as explored by Farooq et al [14]. Their work utilizes stable diffusion models to generate large-scale, high-quality synthetic skin lesion datasets, enhancing machine learning model training by providing a rich variety of realistic dermatoscopic images that improve the robustness and accuracy of skin disease classification models when integrated into training pipelines.

Hair removal in dermoscopic images is a critical preprocessing step in skin lesion analysis. Traditional methods, as reviewed by [15,16], have relied on techniques such as morphological operations and edge detection. However, these methods often struggle with maintaining lesion integrity. The advent of deep learning has introduced new possibilities [17,18,19]. More recent studies have started exploring convolutional neural networks (CNNs) [20,21,22] and Generative Adversarial Networks (GAN) [23,24,25] for this purpose, achieving better accuracy while preserving important dermatological features.

The accuracy of skin disease diagnosis, particularly when diagnosing melanoma, is heavily dependent on the quality of dermoscopic images. As highlighted by [26], combining AI with high-quality images can outperform traditional diagnostic methods. The removal of occluding elements like hair is therefore not just a technical challenge, but a clinical necessity, as emphasized by [27,28]. The integration of recent advanced image processing techniques, such as diffusion models, in this domain holds significant promise for improving diagnostic accuracy and patient outcomes. Moreover, leveraging self-supervised techniques and constructing new datasets could advance the state of the art in this domain. This study addresses these improvement gaps and introduces the *DM–AHR* model that will be described in the next section.

## 3. Proposed Method

**Figure 1:**
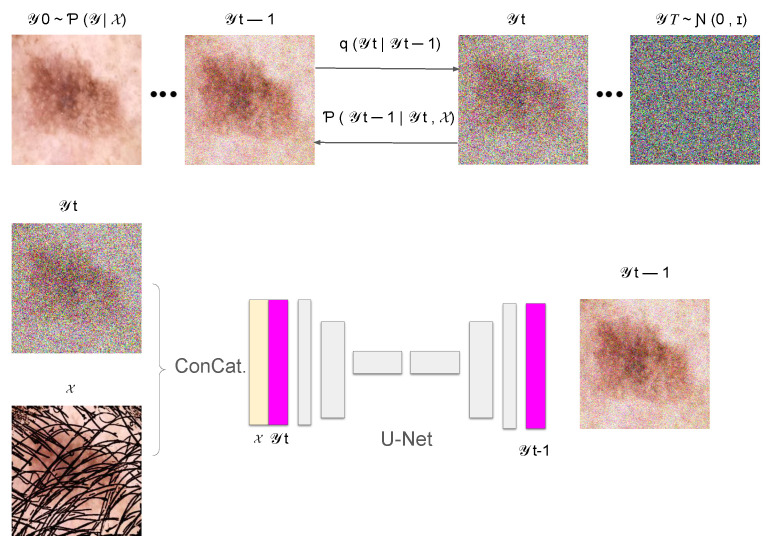
Illustration of the *DM–AHR* Diffusion process.

In this section, we introduce *DM–AHR* (Diffusion Model for Automatic Hair Removal), based on modifying the standard architecture of Denoising Diffusion Probabilistic Models (DDPM) [11] for the purpose of automatic hair removal in dermoscopic images. The global architecture of *DM–AHR* is illustrated in Figure 1.

### 3.1. Dataset and Problem Formulation

Starting from a dataset $D={\left\{x_{i},y_{i}\right\}}_{i=1}^{N}$, where each $x_{i}$ is a dermoscopic image exhibiting hair artifacts, and $y_{i}$ represents the corresponding hair-free image. The dataset is crucial for training the *DM–AHR* model to understand and learn the complex patterns associated with hair in such medical images. Formally, we define our task as learning a mapping function *F*, such that $F\left(x_{i}\right)\approx y_{i}$, where *F* represents the *DM–AHR* model’s transformation capability. Due to the difficulty of constructing real pairs of hairy and hair-free images, the hair is added artificially to real hair-free skin images, as explained in the Experimental Section later.

### 3.2. Conditional Denoising Diffusion Process

The *DM–AHR* model incorporates a sophisticated conditional denoising diffusion process inspired by prominent diffusion model advancements [11,13,29]. The architecture central to this model is a U-Net [30], which is pivotal for denoising and feature differentiation, specifically between hair and skin features in images. This U-Net employs a multi-scale strategy with channels varying in dimensionality corresponding to the resolution of input images, ensuring a tailored approach for different scales, as described in Figure 1.

Although the *DM–AHR* model utilizes the U-Net structure, it is distinctly modified to include a conditioning branch at every denoising step. The inputs to the model are images of skin cancer with hair, which are instrumental in guiding the model to align more accurately with the content of the skin cancer image. This conditioning mechanism significantly enhances the model’s ability to adhere to the relevant features in the images, as visually represented in Figure 1.

The U-Net comprises several layers with depth multipliers at each resolution stage to enhance its capacity and adaptability to the input’s complexity. It utilizes a series of ResNet blocks [31] at each stage, and these blocks help it to learn residual behaviors between noisy and denoised states, thus improving the model’s efficiency and accuracy. Activation functions within these blocks are primarily ReLU, ensuring non-linearity preservation in the denoising process.

For the diffusion process, the model starts with a forward diffusion to generate noisy versions of the original image, which are subsequently refined by the conditional U-Net. This U-Net takes both the noisy image and a low-resolution, interpolated, and up-sampled version of the original image as inputs. It then progressively enhances the image resolution through its depth stages, adapting its parameters dynamically as dictated by the underlying complexity of different image features. The loss function employed is tailored to minimize the difference between the clean and generated images, ensuring the preservation and accurate reconstruction of critical features such as hair and skin textures.

#### 3.2.1. Forward Diffusion Process

The forward diffusion process is modeled as a Markov chain that incrementally adds Gaussian noise to an image. Formally, the process is defined as follows:

(1) $$\begin{array}{cc} q(y_{1:T}|y_{0}) & =\prod_{t=1}^{T}q\left(y_{t}|y_{t-1}\right), \end{array}$$

(2) $$\begin{array}{cc} q(y_{t}|y_{t-1}) & =N(y_{t}|\sqrt{\alpha_{t}}y_{t-1},\left(1-\alpha_{t}\right)I) \end{array}$$

where $y_{0}$ is the original hair-free image, $y_{1:T}$ represents the sequence of images with incrementally added noise, and $\alpha_{1:T}$ are the noise levels at each step. This part is illustrated in the upper part of Figure 1.

#### 3.2.2. Reverse Diffusion Process

In the reverse diffusion process, the aim is to recover the original hair-free image from the noisy image. This is achieved by a learned neural network $f_{\theta }$, which performs denoising at each step. Here, we added the conditioning based on the original image with hair, represented as x, to guide the reconstruction process to remove hair and noise jointly. This is shown in the bottom part of the Figure 1.

The reverse process is defined as follows:

(3) $$\begin{array}{cc} p_{\theta }\left(y_{0:T}|x\right) & =p\left(y_{T}\right)\prod_{t=1}^{T}p_{\theta }\left(y_{t-1}|y_{t},x\right),\\ p(y_{T}) & =N(y_{T}|0,I),\\ p_{\theta }\left(y_{t-1}|y_{t},x\right) & =N(y_{t-1}|\mu_{\theta }\left(x,y_{t},\alpha_{t}\right),\sigma_{t}^{2}I). \end{array}$$

where $\mu_{\theta }$ and $\sigma_{t}^{2}$ are learned parameters of the model and x is the input hair-containing image. The reverse diffusion process is iterative, starting from a purely noisy image and progressively recovering the hair-free image, guided by the input image characteristics.

### 3.3. Training of the Denoising Model

The denoising model $f_{\theta }$, a pivotal component of the *DM–AHR* architecture, is tasked with estimating and eliminating the noise introduced at each step of the forward diffusion process. This process is crucial for reconstructing the hair-free image from its noisy counterpart. The training of $f_{\theta }$ is conducted using pairs of noisy and original hair-free images, with the aim of fine-tuning the model parameters to accurately estimate the noise vector.

#### 3.3.1. Loss Function

The training objective is encapsulated in a loss function that measures the difference between the noise estimated by the model and the actual noise applied during the forward process. Formally, the loss function is as follows:

(4) $$L\left(\theta \right)=E_{(x,y),\epsilon ,t}\left[{∥f_{\theta }\left(x,y_{t},t\right)-\epsilon ∥}^{2}\right],$$

where $y_{t}$ represents the noisy image at time step *t* and ϵ denotes the noise vector. This function aims to minimize the mean squared error between the noise predicted by $f_{\theta }$ and the actual noise.

#### 3.3.2. Training Algorithm

The training algorithm for $f_{\theta }$ is carefully designed to iteratively refine the model parameters, thereby optimizing its ability to estimate and remove noise from images. The process can be dissected into several key steps, each with a mathematical basis, as demonstrated in Algorithm 1:
**Algorithm 1:** Training algorithm for the *DM–AHR* Model $f_{\theta }$ **while** not converged **do**  **for** each batch in the training dataset **do**    Sample a pair $\left(x,y_{0}\right)$ from the batch    **for** t=1 to *T* **do**     Sample noise level $\gamma_{t}$ from a predefined noise schedule     Generate noise $\epsilon_{t}∼N\left(0,I\right)$     Create a noisy image $y_{t}$ using the formula:      $y_{t}=\sqrt{1-\gamma_{t}}y_{0}+\sqrt{\gamma_{t}}\epsilon_{t}$     Compute the loss function for backpropagation:      $L\left(\theta \right)={∥f_{\theta }\left(x,y_{t},t\right)-\epsilon_{t}∥}^{2}$     Update $f_{\theta }$ by applying the gradient descent step:      $\theta \leftarrow \theta -\eta \nabla_{\theta }L\left(\theta \right)$    **end for**  **end for** **end while**

In the training algorithm for the *DM–AHR* model, each iteration commences with sampling a pair of images $\left(x,y_{0}\right)$, where x represents the original image with hair and $y_{0}$ its hair-free counterpart. At each time step *t*, noise is added to $y_{0}$ based on a sampled noise level $\gamma_{t}$, creating a noisy version $y_{t}$. The loss function, which is critical for guiding the model, measures the difference between the model’s estimated noise and the actual noise added. Model parameters are updated via gradient descent, where the learning rate (η) and the gradient of the loss function ($\nabla_{\theta }L\left(\theta \right)$) are key factors. This approach ensures effective learning and robust noise estimation capabilities, enabling the model to accurately map noisy images to their hair-free versions under a variety of noise levels.

### 3.4. Inference Process of the *DM–AHR* Model

During the inference process of *DM–AHR* model, the Iterative Refinement Algorithm [29] is adapted to the current problem. The inference is performed for multiple iterations and guided by the input image containing hair and it leverages the denoising model $f_{\theta }$ (the U-Net model shown in Figure 1) at each iteration.

The algorithm initializes with a noisy image $y_{T}$ and progressively applies denoising to reach the final hair-free image $y_{0}$. The refinement at each step is mathematically governed by the following equation:

(5) $$y_{t-1}=f_{\theta }\left(y_{t},x,t\right)+y_{t},$$

where $y_{t}$ is the noisy image at step *t*, and x is the original hair-containing image. The function $f_{\theta }$ is responsible for estimating the difference between $y_{t}$ and the hair-free image, effectively removing noise and hair artifacts.

The effectiveness of this algorithm in hair removal can be attributed to its iterative nature, allowing gradual refinement of the image. Each iteration removes a portion of the noise and hair, refining the image closer to its hair-free version. The process is described in Algorithm 2, where *T* is the number of iterations fixed by the user to regulate the inference.

**Figure 2:**
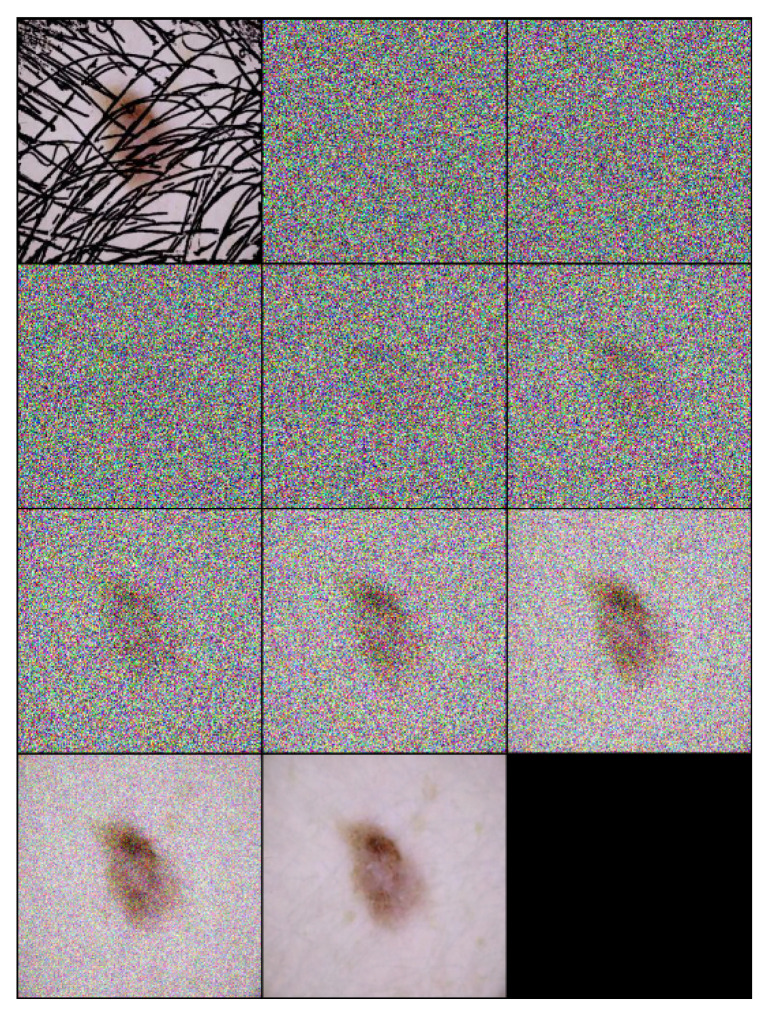
Inference steps of *DM–AHR* applied on one melanoma skin case performed in 10 iterations (T=10).

Each step of the algorithm incrementally removes noise and hair, leveraging the learned model $f_{\theta }$ to guide the transformation towards a clear, hair-free image. An example of the inference process applied on a one sample image is illustrated in Figure 2, and the inference number is 10 (T=10).
**Algorithm 2:** Inference process for hair removal in *DM–AHR*Initialize $y_{T}∼N\left(0,I\right)$**for** t=T down to 1 **do**  Sample noise $\epsilon_{t}∼N\left(0,I\right)$  Apply the denoising model:   $y_{t-1}=f_{\theta }\left(y_{t},x,t\right)+y_{t}$**end for****return** $y_{0}$, the final hair-free image

The noise schedule of the *DM–AHR* model is defined by a piecewise uniform distribution, which is crucial for controlling the noise variance at each diffusion step:

(6) $$p\left(\gamma \right)=\sum_{t=1}^{T}\frac{1}{T}U\left(\gamma_{t-1},\gamma_{t}\right),$$

where $U(\gamma_{t-1},\gamma_{t})$ is a uniform distribution between noise levels $\gamma_{t-1}$ and $\gamma_{t}$, and *T* is the total number of diffusion steps. This noise schedule is vital for the model’s ability to handle various noise levels, enhancing its robustness and adaptability for effective hair removal in medical imaging applications.

### 3.5. Self-Supervised Technique

The training of the *DM–AHR* model employs a novel self-supervised technique, which is specifically tailored for the automatic removal of hair from dermoscopic skin images. This approach leverages the algorithm as shown in Algorithm 3. The key idea is to artificially synthesize hair-like patterns on hair-free dermoscopic images, creating a modified dataset that simulates real-world scenarios in which hair obscures skin lesions.

The process begins with an original, hair-free dermoscopic image, denoted as $I_{original}$. A pre-set number of lines, $N_{lines}$, for example, 1000, are algorithmically drawn on this image to imitate hair. These lines are generated with randomized starting points, lengths, and directions, ensuring a diverse and realistic representation of hair patterns across the dataset.
**Algorithm 3:** Self-supervised technique customized for training skin hair removal models**Input:**  $I_{original}$ {the original hair-free dermoscopic image}**Output:** $I_{modified}$ {Image with the small artificial lines simulating hair-like patterns}$N_{lines}\leftarrow 1000$ {Fix the number of lines to draw}(W,H)← Size of $I_{original}$ {Get the input image dimensions}$I_{modified}=I_{original}$**for** i=1 to $N_{lines}$ **do**     $x_{start}∼U\left(0,W\right)$ {pick a random integer in [0,W]}     $y_{start}∼U\left(0,H\right)$ {pick a random integer in [0,H]}     $L_{length}∼U\left(10,30\right)$ {pick a random integer in [10,30]}     d∼{−1,1} {pick a random direction for diagonal orientation}     $x_{end}\leftarrow x_{start}+d\cdot L_{length}$ {End x-coordinate}     $y_{end}\leftarrow y_{start}+L_{length}$ {End y-coordinate}     Draw line on *I* from $\left(x_{start},y_{start}\right)$ to $\left(x_{end},y_{end}\right)$ on $I_{modified}$**end for****return** $I_{modified}$

**Figure 3:**
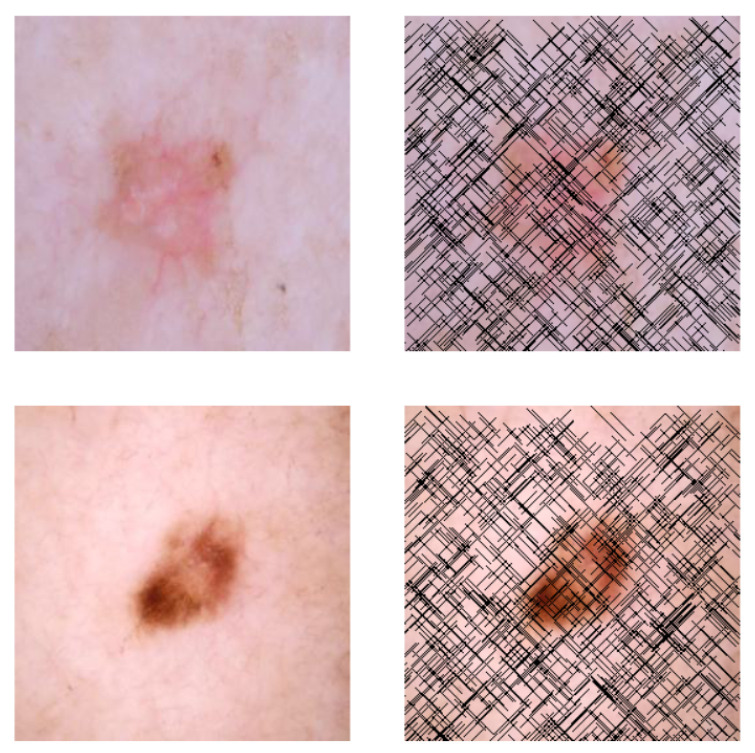
Generated samples using the customized self-supervised technique.

This self-supervised approach significantly augments the model’s proficiency in processing real-world dermatoscopic images while obviating the necessity for a large corpus of manually annotated images obscured by hair. Figure 3 presents two exemplar images produced by this technique, which effectively mimic natural hair patterns. This visual mimicry is instrumental in enabling the *DM–AHR* model to autonomously discern and segregate hair from skin features. Notably, the implementation of this technique has led to a substantial enhancement in the performance of the *DM–AHR* model, which complements its prior training on the meticulously constructed DERMAHAIR dataset, as detailed in the subsequent section.

## 4. Experiments

### 4.1. Experimental Settings

The *DM–AHR* model’s training and evaluation process was conducted using a high-performance NVIDIA Quadro RTX 8000 GPU with 48 GB VRAM, known for its substantial computational capabilities. The *DM–AHR* model underwent an intensive training protocol consisting of 2000 time steps for detailed refinement and 20,000 iterations for comprehensive learning across diverse imaging conditions. This extensive training regime was crucial for iterative improvement in image quality and ensuring the model’s robustness. The Adam optimizer was employed with a fixed rate of 0.0001, facilitating stable and consistent training. The model and resources are made available to the community at: https://github.com/AnasHXH/DM-AHR (accessed on 7 August 2024).

### 4.2. Dataset Description

This study introduces the dataset DERMAHAIR (DERMatologic Automatic HAIR Removal Dataset). It is created to benchmark the task of Automatic Skin Hair Removal to enhance skin diagnosis and analysis. DERMAHAIR was created by sampling 100 images from each of the seven lesion categories within the HAM10000 dataset [32], resulting in 700 unique images. Later, for every image, an artificial hair pattern was picked randomly from the Digital Hair Dataset [33] and added artificially to the image. In the following paragraphs, we will provide a description of the HAM10000 dataset, and we will explain the artificial hair addition process. The DERMAHAIR dataset is open sourced at the following link: https://www.kaggle.com/datasets/riotulab/skin-cancer-hair-removal (accessed on 7 August 2024).

#### 4.2.1. Description of the HAM10000 Dataset

The HAM10000 dataset [32] is a cornerstone in dermatological research, encompassing 10,000 dermatoscopic images that represent a broad spectrum of skin lesions, including melanomas and nevi. This dataset is distinguished by its comprehensive diversity in lesion characteristics and patient demographics. Each image is meticulously annotated with essential metadata such as diagnosis, lesion location, and patient specifics, thereby providing a robust foundation for the development of machine learning models aimed at skin lesion classification and melanoma detection. The HAM10000 dataset includes seven lesion categories: Melanocytic nevi (benign melanocyte tumors, nv), Melanoma (the deadliest form of skin cancer, mel), Benign keratosis (comprising seborrheic keratoses and lentigines, non-malignant growths from keratinocytes, bkl), Basal cell carcinoma (the most common but least aggressive skin cancer, bcc), Actinic keratoses (sun-related precancerous lesions, akiec), Vascular lesions (examples include angiomas, benign vascular proliferations, vasc), and Dermatofibroma (non-malignant fibrous skin tumors, df). The diverse and detailed classification of skin conditions in this dataset renders it invaluable for training advanced AI models in dermatological diagnostics.

We reserved 100 images from each category to form a test set of 700 images, ensuring each class is equally represented in the evaluation process. This stratified sampling minimizes bias and robustly measures the model’s performance across varied conditions. The remaining images were augmented with synthetic hair, forming a diverse training set. Given the specific challenge of hair removal in skin cancer images, the size of the dataset has proven sufficient for achieving high accuracy and reliability in this niche application, as evidenced by our experimental results and the specific adaptations we have made to mitigate the limitations of a smaller sample size.

#### 4.2.2. Description of the Artificial Hair Addition Process

Let us consider a simplified 2D matrix representation of a single color channel from an RGB image:

(7) $$\mathrm{Dermatoscopic}\;\mathrm{Image}\;\left(\mathrm{img}\right)=\left[\begin{array}{cc} 0.3 & 0.6\\ 0.5 & 0.8 \end{array}\right]$$

These matrices symbolize pixel intensity values within a specific color channel, normalized between 0 and 1.

**Hair Mask Images:** To simulate hair artifacts, we employed binary masks sourced from a digital hair dataset [33]. In these masks, hair is represented by white pixels (value = 1), contrasting with the black background pixels (value = 0). Let us consider a simplified representation:

(8) $$\mathrm{Hair}\;\mathrm{Mask}\;\mathrm{Image}\;\left(\mathrm{img}\_h\right)=\left[\begin{array}{cc} 1 & 0\\ 0 & 1 \end{array}\right]$$

**Image Processing and Synthesis:** The process of artificial hair addition encompassed several key steps:

*Normalization of Hair Mask:* Initially, the hair mask image underwent normalization. This process involved inverting the pixel values, effectively converting white hair pixels to black, thus mirroring the appearance of hair in authentic dermatoscopic images:

(9) $$\mathrm{Normalized}\;\mathrm{Hair}\;\mathrm{Mask}=-1\times \left(\mathrm{img}\_h-1\right)=\left[\begin{array}{cc} 0 & 1\\ 1 & 0 \end{array}\right]$$

*Merging Images:* Subsequently, the normalized hair mask was merged with the dermatoscopic image. This crucial step superimposed the artificial hair structure onto the skin lesion image:

(10) $$\mathrm{Merged}\;\mathrm{Image}=\mathrm{img}+\mathrm{Normalized}\;\mathrm{Hair}\;\mathrm{Mask}=\left[\begin{array}{cc} 0.3 & 1.6\\ 1.5 & 0.8 \end{array}\right]$$

*Post-Processing:* The final stage entailed a post-processing routine. By subtracting one from the merged image’s pixel values, the hair regions were effectively restored to their original skin color hues, while normalizing the background:

(11) $$\mathrm{Final}\;\mathrm{Image}=\mathrm{Merged}\;\mathrm{Image}-1=\left[\begin{array}{cc} -0.7 & 0.6\\ 0.5 & -0.2 \end{array}\right]$$

The DERMAHAIR dataset is constructed by picking 100 images from every class in the HAM10000 [32] and constructing a hairy version using this described hair addition process. The generated pair will be used to train any hair removal model, including the *DM–AHR* model described in this study.

In terms of its organization, the dataset is methodically divided into two principal directories: Data_Skin_with_Hair and Data_Skin_without_Hair. Each directory encompasses seven subdirectories corresponding to the seven diagnostic categories, namely akiec, bcc, bkl, df, mel, nv, and vasc. This structure ensures streamlined access and efficient categorization of the dataset.

### 4.3. Evaluation Metrics

The evaluation metrics are divided into two groups. First, there are the metrics used to measure the performance of the *DM–AHR* model itself. Second, there are the metrics used to measure the performance of the skin lesion classification before and after using the *DM–AHR* model to improve the quality of the skin images.

#### 4.3.1. Evaluation Metrics of the *DM–AHR* Performance

In the evaluation of the *DM–AHR* model for Skin Image Dehairing, three key metrics are employed: Peak Signal-to-Noise Ratio (PSNR), Structural Similarity Index Measure (SSIM), and Learned Perceptual Image Patch Similarity (LPIPS).

**Peak Signal-to-Noise Ratio (PSNR)**: PSNR is widely used in image processing to assess the quality of reconstructed images. It is defined as follows:

(12) $$\mathrm{PSNR}=10\cdot \log_{10}\left(\frac{{\mathrm{MAX}}_{I}^{2}}{\mathrm{MSE}}\right),$$

where ${\mathrm{MAX}}_{I}$ is the maximum possible pixel value of the image and MSE is the Mean Squared Error between the original and processed images. Higher PSNR values indicate better quality, with values above 30 dB generally considered good in medical imaging.

**Structural Similarity Index Measure (SSIM)**: SSIM measures the perceived quality of an image by comparing its structural information with that of the original image. It considers changes in texture, luminance, and contrast, and is calculated as follows:

(13) $$\mathrm{SSIM}\left(x,y\right)=\frac{\left(2\mu_{x}\mu_{y}+C_{1}\right)\left(2\sigma_{xy}+C_{2}\right)}{\left(\mu_{x}^{2}+\mu_{y}^{2}+C_{1}\right)\left(\sigma_{x}^{2}+\sigma_{y}^{2}+C_{2}\right)},$$

where x,y are the original and processed images, μ and σ are the mean and variance, $\sigma_{xy}$ is the covariance, and $C_{1},C_{2}$ are constants to stabilize the division. The SSIM values range from −1 to 1, with higher values indicating better similarity.

**Learned Perceptual Image Patch Similarity (LPIPS)**: LPIPS [34] is a metric that evaluates the perceptual similarity between two images, using deep learning models to capture the human visual system’s response. It is defined as follows:

(14) $$\mathrm{LPIPS}\left(I_{1},I_{2}\right)=\sum_{l}\frac{1}{H_{l}W_{l}}\sum_{h,w}\omega_{l}{∥F_{l}{\left(I_{1}\right)}_{h,w}-F_{l}{\left(I_{2}\right)}_{h,w}∥}_{2}^{2},$$

where $I_{1},I_{2}$ are the images being compared, $F_{l}$ denotes the feature map at layer *l*, $H_{l},W_{l}$ are the dimensions of the feature map, and $\omega_{l}$ are layer-wise weights. LPIPS quantifies perceptual differences more aligned with human visual perception.

These metrics collectively provide a multi-faceted evaluation of image quality, which is crucial for accurate diagnosis and analysis in medical imaging.

#### 4.3.2. Evaluation Metrics of the Skin Lesion Classification

After assessing the performance of the *DM–AHR* itself, we move on to the evaluation of the skin diagnosis task before and after using the *DM–AHR*. The classification of skin classes used six key performance metrics: the accuracy, the loss value, the precision, the recall, the specificity, and the F1-score. Each metric provides a unique perspective on the model’s performance before and after using *DM–AHR* to enhance the skin image quality for skin diagnosis purposes.

**Accuracy** is the most intuitive performance measure and it is simply a ratio of correctly predicted observations to the total observations. It is defined as follows:

(15) $$\mathrm{Accuracy}=\frac{\mathrm{Number}\;\mathrm{of}\;\mathrm{Correct}\;\mathrm{Predictions}}{\mathrm{Total}\;\mathrm{Number}\;\mathrm{of}\;\mathrm{Predictions}}$$

where accuracy is particularly useful when the classes in the dataset are nearly balanced.

**Loss**, specifically in the context of neural networks, is a measure of how well the model’s predictions match the actual labels during the training. For the current study, the cross-entropy loss is used in all the experiments:

(16) $$\mathrm{Loss}=-\sum_{i}y_{i}\log\left(p_{i}\right)$$

where $y_{i}$ is the actual label and $p_{i}$ is the model’s predicted probability for that class.

**Precision** is the ratio of correctly predicted positive observations to the total predicted positive observations. High precision relates to the low false-positive rate. It is defined as:

(17) $$\mathrm{Precision}=\frac{\mathrm{True}\;\mathrm{Positives}}{\mathrm{True}\;\mathrm{Positives}+\mathrm{False}\;\mathrm{Positives}}$$

**Recall** (sensitivity) measures the proportion of actual positives that were correctly identified. It is defined as follows:

(18) $$\mathrm{Recall}=\frac{\mathrm{True}\;\mathrm{Positives}}{\mathrm{True}\;\mathrm{Positives}+\mathrm{False}\;\mathrm{Negatives}}$$

**Specificity** is a measure that tells us what proportion of negatives was identified correctly. It complements recall and is defined as follows:

(19) $$\mathrm{Specificity}=\frac{\mathrm{True}\;\mathrm{Negatives}}{\mathrm{True}\;\mathrm{Negatives}+\mathrm{False}\;\mathrm{Positives}}$$

**F1-score** is the weighted average of precision and recall. Therefore, this score takes both false-positives and false-negatives into account. It is a good way to show that a classifier has a good value for both recall and precision. The F1-score is defined as follows:

(20) $$F1-\mathrm{score}=2\times \frac{\mathrm{Precision}\times \mathrm{Recall}}{\mathrm{Precision}+\mathrm{Recall}}$$

Understanding these metrics in tandem allows for a more nuanced interpretation of a model’s performance, particularly in applications like medical image analysis, where the cost of false negatives or false positives can be high.

### 4.4. Performance of the Skin Image Enhancement Using the *DM–AHR* Model

The *DM–AHR* model exhibits high efficacy in enhancing and reconstructing images across various cancer types, as evidenced by its PSNR, SSIM, and LPIPS metrics in Table 1 (PSNR), Table 2 (SSIM), and Table 3 (LPIPS). The *DM–AHR* model performance is compared with the performance of the GAN architecture [35], the SwinIR architecture [36], the standard Stable Diffusion Model (SDM) [37], the *DM–AHR* model without self-supervision (*DM–AHR*), and the full *DM–AHR*(SS) model with self-supervision. The best results are highlighted in bold.

The PSNR values, as shown in Table 1, reveal a consistent superiority of *DM–AHR*(SS), the self-supervised version of *DM–AHR*, over other models. Such high PSNR values indicate that *DM–AHR*(SS) retains maximal fidelity to the original image. This is crucial in medical imaging, where detail preservation is paramount.

Table 2 presents the SSIM scores, underscoring *DM–AHR*(SS)’s ability to maintain structural integrity. The model exhibits exceptional performance in preserving textural and contrast details, as evidenced by it achieving the highest SSIM in every class.

The LPIPS metric, detailed in Table 3, further cements the superiority of *DM–AHR*(SS). LPIPS, as a perception-based measure, highlights the model’s proficiency in maintaining perceptual image quality, a critical aspect that is often overlooked by conventional metrics. The *DM–AHR*(SS) model consistently exhibits the lowest (best) LPIPS scores across all cancer types, confirming its excellence in perceptual image fidelity.

When juxtaposed with models like SRGAN and SwinIR, *DM–AHR*(SS) not only surpasses them in terms of numerical metrics but also in qualitative aspects such as noise reduction, detail enhancement, and overall visual quality. The self-supervised learning approach ingrained in *DM–AHR* (SS) evidently contributes to its nuanced understanding and handling of complex dermatoscopic images.

Table 4 shows an example of a skin image without hair, the artificially added hair, and the reconstructed image using the *DM–AHR* model. We can see visually that the image is reconstructed at a high fidelity scale, alongside its histogram.

In Table 5, we show samples from different skin lesion classes with the reconstructed images using the *DM–AHR* model. We can also see the high reconstruction quality of the model and its successful discrimination between the skin features and the hair degradation patterns.

### 4.5. Improvement of the Medical Skin Diagnosis after Using *DM–AHR*

In the evolving landscape of medical skin diagnosis, the robustness and accuracy of skin cancer classification models are paramount. This study presents a comprehensive evaluation of three state-of-the-art deep learning architectures: resnext101_32x8d [38] (in Table 6), maxvit_t [39] (in Table 7), and Swin-Transformer [40] (in Table 8). The performance of these models is assessed based on the original images, on the hairy version of the images, and finally on the dehaired version of the images using *DM–AHR* model. The ensuing tables encapsulate key performance metrics, including accuracy, loss, precision, recall, specificity, and F1-score for each model under the specified conditions.

For the resnext101_32x8d model, the introduction of hair to the images led to a substantial decrease in accuracy, from an impressive 98.5% in the original state to 87.9% post-modification. This significant decline highlights the model’s sensitivity to visual perturbations. However, the subsequent application of the IDM hair removal method effectively mitigated this impact, elevating the accuracy to 97.3%. The precision and recall metrics mirrored this trend, suggesting that *DM–AHR* preprocessing can significantly recover the model’s discriminatory power.

Similarly, the maxvit_t model, while demonstrating remarkable resilience with an original accuracy of 98.9%, also experienced a decline in performance after hair was added to the image, though to a lesser extent (95.1% accuracy). Post-*DM–AHR* application, the model not only recovered but exceeded its original performance, achieving an accuracy of 99.3%. This result suggests that the IDM method may not only compensate for the added noise but also enhances the model’s overall interpretive accuracy. This is a notable property of the *DM–AHR* model that is worthy of investigation in future research studies.

**Figure 4:**
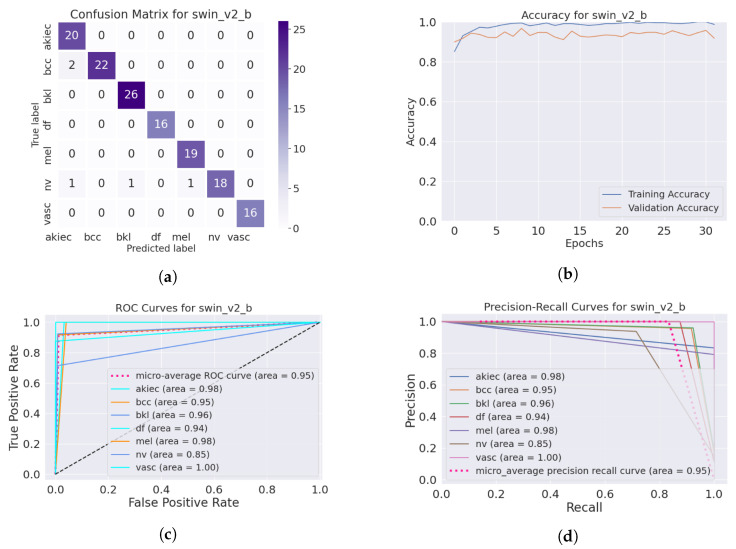
Performance of the skin classification using Swin Transformer before using *DM–AHR* (hairy occluded images): (**a**) Confusion matrix, (**b**) accuracy curve during training, (**c**) ROC curve, and (**d**) precision–recall curve.

**Figure 5:**
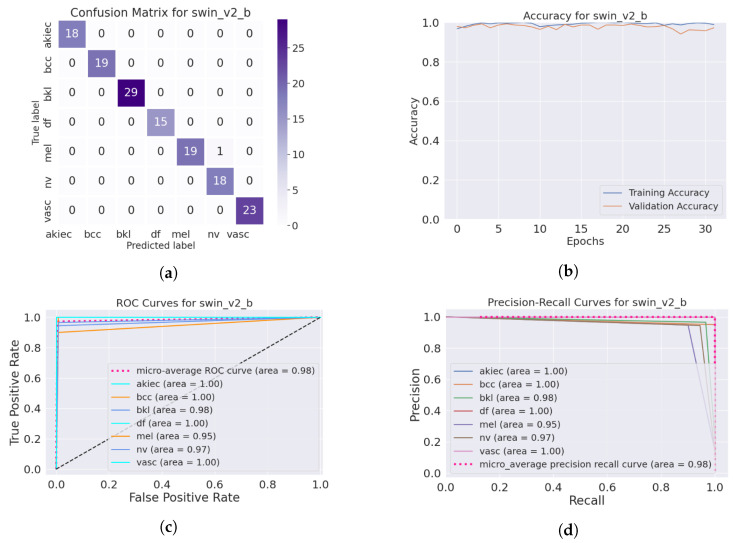
Performance of the skin classification using Swin Transformer after using *DM–AHR* (dehaired images): (**a**) Confusion matrix, (**b**) accuracy curve during training, (**c**) ROC curve, (**d**) precision–recall curve.

The SwinTransformer model showed a consistent pattern of performance reduction upon hair addition, followed by a notable recovery with IDM processing. The original accuracy of 98.3% reduced to 96.8% after hair was added and then increased to 99.3% post-*DM–AHR*, paralleling the trends seen in the other models. This consistency across different architectures underscores the general applicability and effectiveness of the *DM–AHR* preprocessing technique in enhancing model robustness. Figure 4 and Figure 5 illustrate the performance of the skin classification and diagnosis using the Swin Transformer before and after the enhancement step using the *DM–AHR* model. The analysis of the confusion matrices, the accuracy curves, the ROC curves, and the precision–recall curves shows the significant improvement of the skin analysis after the preprocessing using the *DM–AHR* model.

Concerning the comparative study between these three skin classification models, this study suggests distinct applications for each model based on their performance. The resnext101 model is ideal for environments with clean, structured data due to its high precision and accuracy in such conditions. The maxvit_t model, which demonstrates remarkable robustness to occlusions, is recommended for more challenging clinical settings where data variability and robustness are critical. SwinTransformer, with its consistent performance across varying conditions, serves as a versatile option for diverse medical imaging tasks. In scenarios requiring robust preprocessing, both maxvit_t and SwinTransformer show significant improvements, making them suitable choices. A combination of these models or ensemble techniques could be advantageous in environments with diverse and unpredictable image qualities, leveraging the individual strengths of each model. Table 9 shows the training time and the model size for every model apart.

In the deployment of the *DM–AHR* model, its primary function is to preprocess dermoscopic images by removing hair before classifying the type of skin cancer. While the model achieves high accuracy, there are instances of misidentification, albeit at a very low rate. These rare errors typically involve the misclassification between hair artifacts and certain types of skin lesions. However, it is important to highlight that in a clinical setting, the final diagnosis is corroborated by expert dermatologists. The *DM–AHR* model significantly aids this process by enhancing image clarity, thus making the detection of skin anomalies more straightforward and rapid. This not only assists dermatologists in making more informed decisions but also contributes to quicker diagnostic processes, potentially speeding up the initiation of appropriate treatment plans and improving patient outcomes. The integration of the *DM–AHR* model into clinical workflows is therefore seen not just as a technological enhancement but as a vital tool in the fight against skin cancer, offering a blend of precision and speed that is crucial for effective medical care.

The collective findings from this study highlight the critical role of preprocessing in medical image analysis. Introduced herein, the *DM–AHR* model has demonstrated notable efficiency in mitigating a common form of medical image degradation, underscoring its potential utility as a preprocessing tool not only for dermatological assessments but also across various medical imaging applications. However, it is important to note that the computational demands of the *DM–AHR* model are considerable. Specifically, processing an image of size 256×256 pixels through 2000 steps requires approximately 2 min on a single GPU RTX 8000 with 48 GB of VRAM. This substantial resource requirement is justified by the model’s capability to effectively remove hair from dermoscopic images, which is a crucial step in enhancing the visibility and detectability of skin cancer lesions. Skin cancer, as a potentially lethal disease, necessitates the highest accuracy in diagnostic imaging processes.

The *DM–AHR* model, therefore, presents a trade-off between computational expense and clinical value. The increased processing time and resource use are offset by the model’s ability to deliver clearer, more interpretable images that are essential for accurate diagnosis and subsequent treatment planning. This balance underscores the model’s practical relevance, particularly in settings where diagnostic precision is paramount.

## 5. Conclusions

In conclusion, the *DM–AHR* model presents a significant contribution to the topic of dermatological image processing. The model, with its self-supervised learning approach, has demonstrated remarkable proficiency in enhancing skin lesion images, thereby facilitating more accurate and efficient skin diagnostics. With skin cancer rates increasing globally, tools like *DM–AHR* can play a major role in early detection and treatment, possibly saving lives. The model’s ability to effectively remove hair from dermatoscopic images addresses a significant challenge in the field, paving the way for clearer and more reliable lesion analysis. However, the high processing cost of *DM–AHR* remains its main limitation that should be addressed in future research works.

Future research should focus on integrating the *DM–AHR* model into clinical workflows to harness its diagnostic capabilities in real-world settings and extend its application to other medical imaging modalities such as ultrasound, CT, and MRI. Exploring the adaptation of diffusion-based models to address various imaging artifacts could lead to significant development in medical image analysis by improving diagnostic accuracy, expediting disease detection, and enhancing patient care. This integration promises comprehensive pre-processing tools that support a wide range of diagnostic procedures, potentially transforming patient outcomes by facilitating early disease detection and treatment through advanced technological applications.

## Figures and Tables

**Table 1:** Comparison of the PSNR performance of *DM–AHR* with other models.

Skin Cancer Type	SRGAN	SwinIR	SDM	*DM–AHR*	*DM–AHR*(SS)
Actinic keratosis	32.401835	39.401370	33.731562	39.095776	**41.280412**
Basal cell carcinoma	31.700830	39.625874	34.317885	38.633978	**40.806729**
Benign Keratosis	31.644829	39.894135	34.109435	39.228128	**41.026910**
Dermatofibroma	31.807330	39.207628	34.196231	39.148305	**40.910219**
Melanoma	30.917425	38.493721	33.536934	38.728289	**39.883010**
Melanocytic nevi	32.242417	39.974818	34.960045	39.091819	**41.212679**
Vascular lesions	32.633316	41.737362	35.790086	39.963068	**42.170609**
**Average**	31.906854	39.762129	34.377454	39.12705	**41.041512**

**Table 2:** Comparison of the SSIM performance of *DM–AHR* with other model.

Skin Cancer Type	SRGAN	SwinIR	SDM	*DM–AHR*	*DM–AHR*(SS)
Actinic keratosis	0.881940	0.959481	0.865522	0.960036	**0.967910**
Basal cell carcinoma	0.882850	0.957559	0.872703	0.955464	**0.964175**
Benign Keratosis	0.879369	0.959514	0.871176	0.956925	**0.965370**
Dermatofibroma	0.869542	0.957726	0.865824	0.956125	**0.964412**
Melanoma	0.868869	0.953418	0.860095	0.951718	**0.961027**
Melanocytic nevi	0.879389	0.955402	0.892593	0.954957	**0.962382**
Vascular lesions	0.899865	0.968740	0.917110	0.967328	**0.973995**
**Average**	0.880261	0.958834	0.87786	0.957508	**0.965613**

**Table 3:** Comparison of the LPIPS performance of *DM–AHR* with other models.

Skin Cancer Type	SRGAN	SwinIR	SDM	*DM–AHR*	*DM–AHR*(SS)
Actinic keratosis	0.171810	0.042776	0.050473	0.018420	**0.012620**
Basal cell carcinoma	0.175114	0.038973	0.046981	0.020546	**0.013838**
Benign Keratosis	0.185645	0.036158	0.047129	0.020156	**0.013638**
Dermatofibroma	0.182939	0.042536	0.045433	0.020481	**0.013376**
Melanoma	0.192164	0.045766	0.049873	0.023361	**0.015010**
Melanocytic nevi	0.164768	0.040105	0.036954	0.019475	**0.012884**
Vascular lesions	0.131372	0.025131	0.027423	0.013758	**0.008439**
**Average**	0.171973	0.038777	0.043467	0.019457	**0.012829**

**Table 4:** Visual results of *DM–AHR* applied on one set of skin images with the corresponding histograms.

	Original Skin Image	Skin Image with Hair	Skin Image after *DM–AHR*
Result	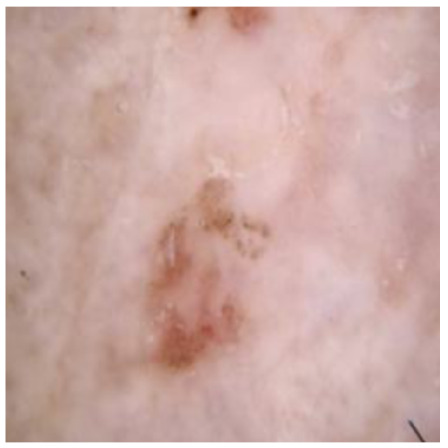	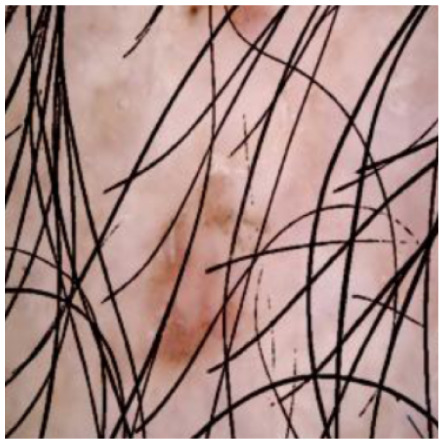	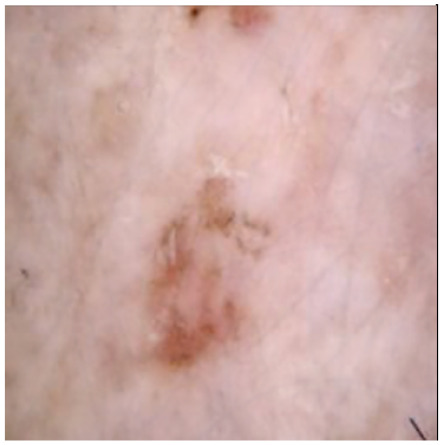
Histogram	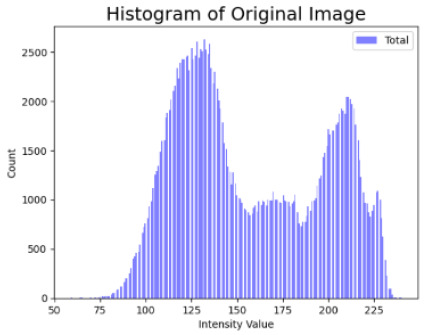	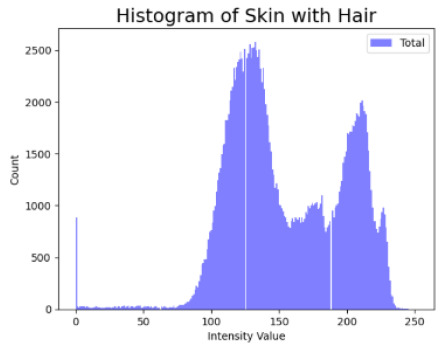	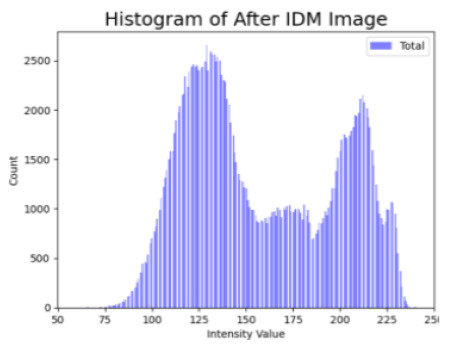

**Table 5:** Comparison of skin images before and after application of *DM–AHR* dehairing.

Cancer Type	Original	After Hair	After *DM–AHR*
Actinic keratosis	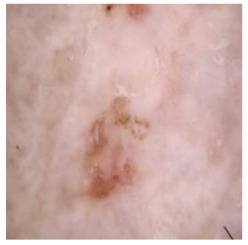	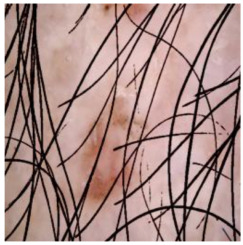	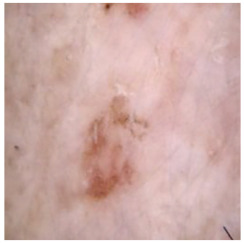
Basal cell carcinoma	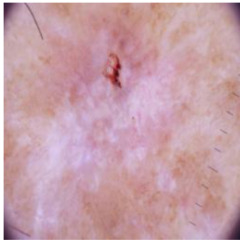	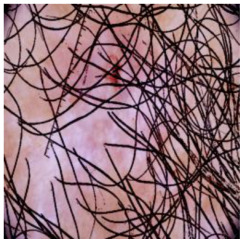	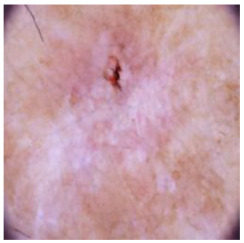
Benign keratosis	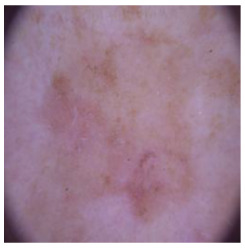	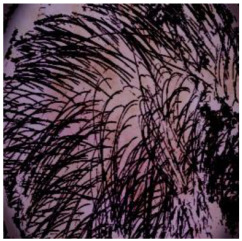	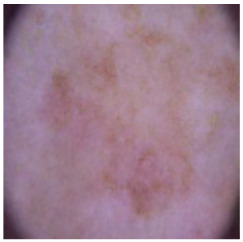
Dermatofibroma	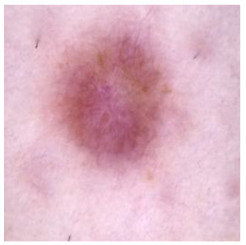	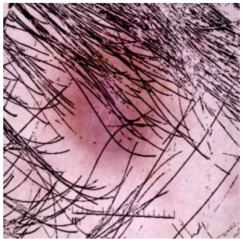	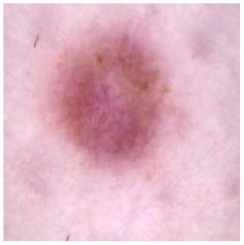
Melanoma	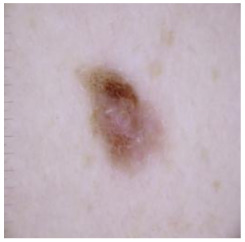	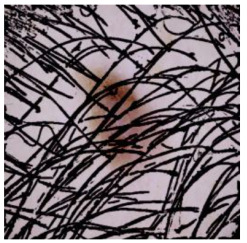	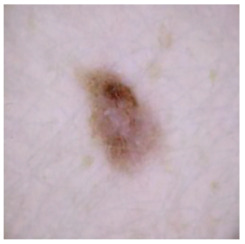
Melanocytic nevi	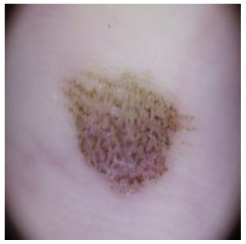	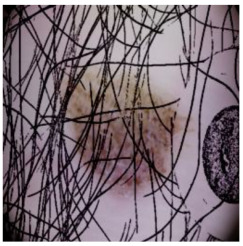	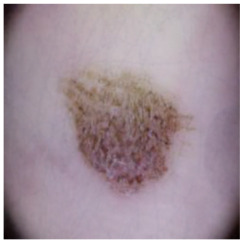
Vascular lesions	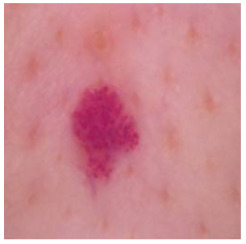	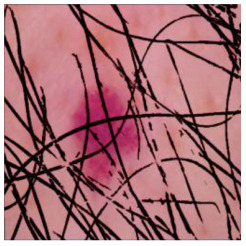	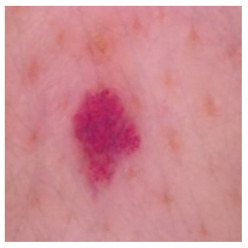

**Table 6:** Performance metrics for the resnext101_32x8d model.

Metric	Original	After Adding Hair	After *DM–AHR* Hair Removal
Accuracy	0.985242	0.879514	0.972731
Loss	0.050478	0.565113	0.099951
Precision	0.9856	0.8624	0.9825
Recall	0.9852	0.8795	0.9727
Specificity	0.9973	0.9778	0.9964
F1-score	0.9853	0.8660	0.9766

**Table 7:** Performance metrics for the maxvit_t model.

Metric	Original	After Adding Hair	After *DM–AHR* Hair Removal
Accuracy	0.988786	0.950656	0.992857
Loss	0.042649	0.168300	0.024280
Precision	0.9889	0.9436	0.9940
Recall	0.9888	0.9507	0.9929
Specificity	0.9980	0.9906	0.9988
F1-score	0.9888	0.9462	0.9933

**Table 8:** Performance Metrics for the SwinTransformer Model.

Metric	Original	After Adding Hair	After IDM Hair Removal
Accuracy	0.983420	0.967687	0.992957
Loss	0.066955	0.114440	0.027340
Precision	0.9837	0.9689	0.9925
Recall	0.9834	0.9677	0.9929
Specificity	0.9970	0.9941	0.9988
F1-score	0.9834	0.9665	0.9925

**Table 9:** Model size and training time for different models.

Model	Model Size (MB)	Training Time
resnext101_32x8d	1340.72	403 m 32 s
maxvit_t	834.43	196 m 23 s
SwinTransformer	911.96	418 m 49 s

## Data Availability

Data Availability Statements at https://www.kaggle.com/datasets/riotulab/skin-cancer-hair-removal (accessed on 7 August 2024).
